# The Biomechanisms of Metal and Metal-Oxide Nanoparticles’ Interactions with Cells

**DOI:** 10.3390/ijerph120201112

**Published:** 2015-01-22

**Authors:** Sondra S. Teske, Corrella S. Detweiler

**Affiliations:** Department of Molecular, Cellular and Developmental Biology, University of Colorado Boulder, MCD Biology, Campus Box 347 UCB, Boulder, CO 80309, USA; E-Mail: detweile@colorado.edu

**Keywords:** biomechanism, metal-oxide, nanoparticle, nanotoxicology, QSAR model

## Abstract

Humans are increasingly exposed to nanoparticles (NPs) in medicine and in industrial settings, where significant concentrations of NPs are common. However, NP interactions with and effects on biomolecules and organisms have only recently been addressed. Within we review the literature regarding proposed modes of action for metal and metal-oxide NPs, two of the most prevalent types manufactured. Iron-oxide NPs, for instance, are used as tracers for magnetic resonance imaging of oncological tumors and as vehicles for therapeutic drug delivery. Factors and theories that determine the physicochemical and biokinetic behaviors of NPs are discussed, along with the observed toxicological effects of NPs on cells. Key thermodynamic and kinetic models that explain the sources of energy transfer from NPs to biological targets are summarized, in addition to quantitative structural activity relationship (QSAR) modeling efforts. Future challenges for nanotoxicological research are discussed. We conclude that NP studies based on cell culture are often inconsistent and underestimate the toxicity of NPs. Thus, the effect of NPs needs to be examined in whole animal systems.

## 1. Introduction

Nanoparticles (NPs) are defined as having at least one dimension measuring 100 nanometers or less [1]. NPs modulate a wide range of biological reactions, including inflammation, cell toxicity, and genotoxicity [2,3]. Since nanomaterials can bind to a wide variety of substances, including proteins, drugs and nucleotides, and are often engineered to target specific organs and tissues, they can provide substantial benefits for nanomedical applications. One example is cancer treatment, where one type of NP enables tumor imaging and other types deliver chemotherapy to those same pathologic tissues based on their selective cytotoxicity towards specific cancer cell lines [1]. Biomolecules are altered by NPs through energy exchanges within specific thermodynamic, kinetic and physicochemical boundaries [4,5]. Multiple factors affect the reactivity of soluble NPs with biomolecules, including NP size, core composition, shape, surface properties, purity and manufacturing method. In addition, solute conditions, such as pH, ionic strength, and the presence of proteins and other biomolecules affect NP stability, aggregation, agglomeration and reactivity with host biomolecules [6]. The purpose of this review is to present proposed theoretical mechanisms of NP modification of biomolecules and to discuss the challenges in comparing and modeling NPs effects on cells.

## 2. General Aspects of Nanoparticles

### 2.1. Many Properties of NPs Differ from That of Their Larger Counterparts

The study of nanomaterials is a nascent science and a systematic characterization of NPs remains to be done. The application of NPs for biomedical use began in 1995 with the introduction and U.S. federal approval of a drug encased in a liposome (DOXIL, doxorubicin) designed to treat AIDS-related Kaposi’s sarcoma. This NP, like most NPs used in biomedicine and industry, is considered a colloid because it is suspended in a liquid medium [7]. However, colloid research of the last 20 years is only partially able to predict NP behavior because NPs violate a key assumption of the equations that define colloidal behavior; their small size creates such curvature of the surface that it cannot be considered flat, an essential assumption of one of the equations that models and predicts colloidal behavior [5]. Data derived from colloidal particles (usually ranging in size from nanometers to micrometers in at least one linear direction) have been used to develop the equations comprising the two accepted models of colloid behavior, the Derjaguin-Landau-Verwey-Overbeek (DLVO) and the Extended DLVO theories (XDLVO) [4,5]. DLVO and XDLVO predict colloid behavior based on a combination of seven different attractive and repulsive forces, such as van der Waal’s, and the Electrostatic Double Layer (EDL). The combination of these forces determines the net potential energy distribution over the separation distance between particles and whether the particles in solution remain dispersed or agglomerate. Unlike larger particles, NP surfaces have considerable curvature and small radii, which means that a greater percentage of the atoms make up the NP are on the surface. This property affects NP electronic structure, surface charge behavior and reactivity, all of which affect aggregation. Until assumptions in DLVO and XDLVO can be revised to account for the cumulative exertions of the seven forces acting on NPs, we will not be able to predict the chemical reactivity and behavior of NPs based on the data derived from testing larger particles of the same chemical. 

### 2.2. EDL, Zeta-Potential and NP Coagulation in Solution

It has emerged that zeta-potential (an approximate measure of the Electrostatic Double Layer (EDL)) may be a reliable empirical indicator of NP coagulation and reactivity in solution. Zeta potential is a measure of the total electric potential of all ions and particles in solution, and therefore is affected by changing the pH, or ionic strength. Zeta-potential measurements range from 0 to ±60 mV. High readings (>±30 mV) suggest increased stability due to increased electrostatic repulsion. Lower readings (<±30 mV) indicate a tendency to coagulate. At the isoelectric point (0 mV), electrostatic repulsive forces are lowest and coagulation is most likely. If a solution containing NPs shifts to a lower ionic strength, then the zeta potential will increase because the EDL expands to balance electrostatic repulsive and attractive forces and the NPs tend to disperse. If there are too many ions, then the zeta potential decreases, and NPs may aggregate and precipitate. Thus, the propensity of NPs to coagulate can be reduced by the addition of acids or bases, or by decreasing the ionic strength of the solution and thereby increasing the zeta-potential [5]. These observations have practical implications. For instance, zebrafish embryos are extremely sensitive to gold NPs under conditions of low ionic strength, in which the NPs disperse, but not high ionic strength [8]. 

### 2.3. NP Characteristics That Affect Behavior: Size

Coagulation (reactivity with other molecules) is impacted by several characteristics of NPs, most importantly their size. Empirical testing with NPs demonstrated that smaller sized NPs with correspondingly larger surface areas produce enhanced inflammatory and cytotoxic responses in cells and organisms. High surface area/volume ratios promote agglomeration through weak bonds and aggregation through stronger bonds. In mouse macrophage-like cells, NPs with diameters that were only 2–3-fold smaller than larger control NPs induced cytotoxicity and promoted macrophage production of pro-inflammatory modulators, such as reactive oxygen species (ROS), cyclooxygenase-2 (COX-2) and cytokines (TNF-α). NPs examined in these assays included the following: silver (15 nm, 40 nm), aluminum (20 nm, 50 nm), carbon-coated silver (25 nm, 45 nm), carbon black (20 nm, 40 nm), and gold (20 nm, 40 nm) [9]. In another study with rat pulmonary alveolar macrophages, increased surface area of titanium dioxide NPs increased production of reactive nitrogen species (NO) and TNF-α [5]. For some NPs, agglomeration reduces reactivity by masking reactive sites [5]. However, in general, smaller NPs are more pro-inflammatory or toxic because their increased surface area to volume ratio increases electrochemical reactivity.

### 2.4. NP Characteristics That Affect Behavior: Shape, and Crystallinity

NPs are not necessarily spherical, in which case axis orientation strongly influences the tendency to coagulate, as particles maximize the attractive forces over the shortest distance. Aggregation and hence, inflammation are most likely to occur with NPs that consist of thin sheets of minerals (platelets), followed by rods, cylinders, and spheres [5]. For instance, increasing aggregation of hematite particles correlates with decreasing diameter due to decreasing repulsive EDL forces [10]. The effect of crystallinity on agglomeration and aggregation is demonstrated by titanium dioxide NPs. The anatase form of titanium dioxide has a more anisometric crystal arrangement than the rutile form, and this characteristic correlates with enhanced genotoxic and inflammatory responses [3,5,11]. 

### 2.5. NP Characteristics That Affect Behavior: Surface Properties

Surface characteristics affect how NPs react with other biological entities in solution through the same attractive and repulsive electrochemical forces that are key to DLVO and XDLVO theory. In particular, hydrophobicity, surface charge, and charge distribution have been demonstrated to influence NP fate and behavior in an organism. 

Hydrophobicity of the NP surface promotes agglomeration, preferential uptake by cells, and biological reactivity. Studies of fullerenes with graded lipophilicity in their shells show that those with a higher proportion of embedded hydroxyl and aliphatic groups are less cytotoxic because they produce fewer oxygen radicals [12,13]. Iron oxide NPs alone are highly hydrophobic, whereas those designed with certain surface functional groups can be less hydrophobic and less toxic. As NP hydrophobicity increases, the propensity to bind host proteins increases along with increased uptake by macrophages [14]. To prevent phagocytosis of NPs and to increase NP access to the target organ, hydrophilic groups, such as PEG polymers, are often added to the NP surface [15]. Thus, hydrophobicity of the surface of NPs can be modulated to produce the desired biological outcome.

The type, distribution, and density of charge of the NP surface are strong determinants of cytotoxic interactions. For example, differences in the surface charge of gold and silver NPs determine the effect of NPs on the integrity of the cell membrane and on ATP production in Gram-negative bacteria [16,17]. Baby hamster kidney (BHK21) and human colon adenocarcinoma (HT29) cultured cells also exhibited membrane damage upon exposure to silver NPs, as elucidated by atomic force microscopy. NPs promoted the accumulation of bundles of membrane proteins in the lipid bilayers, including pits and protrusions of the cell surface and the triggering of a signaling cascade that led to apoptosis [18]. Analysis using a constructed lipid bilayer simulation model suggests that the differential distribution and fate of positive and negatively charged gold NPs is controlled by the electrostatic interaction of the ligand termini of the NP surface with lipid bilayer head groups. Anionic and some cationic gold NPs are absorbed onto the bilayer membrane and endocytosed. Cationic NPs are internalized five times more frequently than anionic NPs. Approximately half of cationic gold NPs are endocytosed whereas the other half likely diffuse into cells via disruptions in the membrane. Disruption of the membrane and cell entry by cationic gold NPs escalates with increased charge densities [19]. The differential fates of neutral, anionic and cationic charged ligands attached to gold NPs were explored in a zebrafish model. Neutral ligand Au-NPs had no adverse effects, whereas positively and negatively charged ligands had distinct effects. Positively-charged ligand-Au NPs had longer residence times and increased mortality at low doses. Negatively-charged ligand-Au NPs were eliminated rapidly but induced malformations [20]. 

Surface defects inherent to the synthesis of the NP can also cause significant damage to exposed organisms. One study related the degree of toxicity to the concentration of strained three-membered rings (3MRs) of surface silanol (silica-hydroxyl) groups that generated hydroxyl radicals (OH^•^) through a Fenton-like reaction in amorphous silica [21]. Strained 3MRs are molecules that have bonds that form at abnormal angles, an unstable condition. Differing methods to manufacture amorphous silica produce diverse surface electrochemical properties. Fumed silica is produced at high temperatures between 1200–1400 °C with subsequent rapid quenching. In contrast, colloidal (Stöber) silica is created at a lower temperature through condensation of silanol groups to yield anhydrous silica oxide (siloxane). Both manufacturing methods generate siloxane frameworks and hydrogen-bonded silanol groups, but fumed silica has a higher proportion of strained 3MRs and chain-like aggregates that generates hydroxyl radicals, not observed with colloidal silica. Fumed silica induces cell membrane damage, causing intracellular calcium channel influx and loss of mitochondrial membrane potential. These membrane perturbations had pro-inflammatory effects, specifically Nalp3 inflammasome activation and secretion of the IL-1β cytokine in cell culture studies [21]. The influence of the specifics of the industrial method to produce similar NPs with radically different surface properties and inherent toxicology cannot be underestimated. Because of these differences, a primary goal of a joint government‒nanomaterials industry initiative is to establish standard, benchmarked manufacturing methods for nanomaterials in order to reduce toxicological hazards [22]. 

### 2.6. Protein Coronas form on NPs in vivo and Have Both Beneficial and Adverse Effects

Soluble proteins can interact with NPs to form a halo (“corona”) that affects NP activity. The specific composition of protein coronas vary based on size and surface properties of the NP, and coronas develop over time until they reach equilibrium [23]. Protein coronas have two layers, a permanently adsorbed layer, or “hard corona” nearest the NP surface and a more distal “soft corona” composed of reversibly adsorbed materials [24]. Researchers have reported some beneficial effects of protein coronas; coronas can overcome attractive aggregative forces and thereby prevent aggregation [25] and reduce NP mediated inflammatory toxicity [5]. In addition, a protein corona can specifically interact with biomolecules in a biologically relevant way. For example, carbon nanotubes and silica NPs adsorb albumin, which blunts LPS-induced expression of the pro-inflammatory mediator Cox-2 [26]. Albumin binding on the surface of these same NPs promotes internalization into tumors and endothelial cells. 

It has been shown that the hard corona compositions for many NPs tested *in vitro* vary over hours to days and are most likely not in equilibrium, as compared to the rapid fluxes of the soft corona that occur within seconds. Hard coronas are relatively stable, with substantially slower biomolecule adsorption kinetics than soft coronas. Moreover, hard coronas retain biomolecules and therefore have molecular memories of previous environments. For example, lung surfactant proteins are present in the hard corona of inhaled NPs even after they enter bloodstream [27]. This observation suggests that the biodistribution and biokinetics of nanomaterials in the human body may develop over longer time periods than are typically observed in standard laboratory experiments. 

In cell culture experiments and in the body, NPs encounter a diversity of proteins, lipids, and sugars which are reflected in the composition of most hard coronas [28]. However, surveys of NPs *in vivo* demonstrate that mature protein coronas are predominantly constituted of apolipoproteins [23,24] which are recognized by multiple cell surface receptors and allow entry into many cells. For example, coronas with apolipoprotein E (ApoE) have increased access to the brain [24]. Solid lipid NPs with surface coatings of polyethylene oxide (PEO) monomer chains and coronas of ApoE coronas crossed the blood brain barrier at rates that increased with increasing ApoE [29]. In addition, enhanced binding of ApoE to the surfaces of NPs with other surface compositions, including drugs, facilitates transport across the barrier into the brain [14]. Thus, protein binding is an important determinant of biodistribution, pharmacokinetics, and toxicity for NPs [14].

In recent *in vitro* studies, the critical role of serum concentration as a primary modulator of toxicity to NPs has been documented. High serum protein concentrations allow for protein attachment to hard coronas, which shields cells from immediate damage after engulfment of NPs [30,31]. *In vivo* serum concentrations are 10 to 50-fold higher than those used in cell culture (2%–10%, approximately 4 mg/mL) [30]. Thus, NP targeting of cells in culture is not necessarily reproducible *in vivo* [32]. In one example, the cytotoxic effects of NPs on A549 (human adenocarcinoma alveolar basal epithelial) cells decreased with increasing concentrations of serum, from 4 to 40 mg/mL. Cytoxicity was evaluated based on cell death and decreased cell proliferation and ATP levels. The highest concentrations of serum fully protected A549 cells from NP toxicity [32]. The same investigators in related studies with lower concentrations of serum determined that NPs induce damage in a variety of different cell types via apparently similar mechanisms [30,31,32,33]. Endocytosis of NPs leads to degradation of the hard corona and exposure of NP-ligand surfaces in the lysosome. Depending upon the extent of damage incurred during lysosomal protease activity, depolarization of the mitochondria can occur and trigger apoptosis and the caspase cascade, ending with possible nuclear degradation and rupture of the plasma membrane. Besides low serum levels not providing enough cellular protection, damage in the lysosome is incurred because some exposed NP-ligand surfaces are toxic. For instance, amino groups, can become protonated under the acidic conditions of the lysosome, and propagate damage. Quaternary ammonium groups, in contrast, remain cationic and are minimally harmful [31]. Thus, NP properties in combination with experimental and cellular microenvironments determine the biological outcome of NP engulfment. 

NP protein coronas can also interfere with protein folding, enzymatic activity, and can enhance cross-linking and fibrillation (fiber formation). How NP coronas mediate these effects is undetermined [34,35]. Silica dioxide NPs have been reported to inhibit normal enzymatic functions and are cytotoxic [36]. SiO_2_ NPs also decrease α-helical structure and enzymatic activity of lysozyme [37]. A similar report of substantial loss of lysozyme function and transformation from an α-helical structure to a β-sheet has been attributed to titanium dioxide NP exposure [38]. In contrast, lysozyme α-helical content increased upon exposure to ZnO NPs [39]. The characteristics of NPs that interfere with normal protein folding and function are unknown and testing for their effects remains complex.

There is evidence that inappropriate secondary folding of proteins/peptides induced by NP coronas might increase the prevalence of severe dysfunctional amyloid diseases (such as Alzheimer’s, and Creutzfeld-Jacob) that form insoluble fibrils [40,41]. NPs are implicated in the transformation of an initially soluble peptide into an amyloid fibril which involves a transitional partially unfolded protein intermediate. NPs initiate nucleation of the fibril and reduce the kinetic rate-limiting step of nucleation in the lag phase. This involves an endothermic process of laying down the initial fibril layer onto the NP, with subsequent multiple binding and dissociative events in the lag phase to form several protein layers that will promote oligomer growth. NPs also enhance the proportion of fibrils that self-assemble after nucleation due to augmented protein concentrations adsorbed on the high surface area of the NP as compared to larger particles. The specific molecular mechanisms are unknown, but the coronas of NPs also increase fibril growth [40]. Similar findings were reported for silicon dioxide (SiO_2_) NPs that induced protein aggregation in the nucleoplasm, resulting in protein aggresomes and neuronal intranuclear inclusions co-localized with topoisomerase I in a pathology that resembled that of expanded polyQ neurodegenerative disorders such as Huntington’s disease [36]. 

In summary, NPs may change the properties of proteins and thereby pose a critical danger to exposed organisms. Moreover, the injurious consequences may not be readily discernible in cell culture assays, but rather only be detectable in long-term developmental and generational *in vivo* studies. Determining which critical elements of these complex environments are essential to include in nanomaterial testing and which instruments can easily quantify them through a standardized method is the ongoing challenge. Adsorption kinetics of proteins on NPs have been analyzed using infrared, fluorescence correlation, UV, fluorescence and Raman spectroscopy, and surface plasmon resonance. Protein conformation also has been quantified by NMR circular dichroism, enzyme activity assays, and infrared spectroscopy. The structure of the corona-NP conjugation has been analyzed through microscopy (TEM and AFM) [30]. Adsorption indices have been successfully used to model nanotoxic effects [42]. Comparison of all these approaches along with new techniques will hopefully provide new insights into quantifying the relevance of environmental biomolecules in nanotoxicology.

### 2.7. Ligands Attached to Metal and Metal-Oxide NPs Alter Functioning

Bioengineered attachment of non-protein ligands to NP surfaces provides biotolerance, stabilization, functionality, and/or anchor sites for functional groups. Ligands can be multifunctional, and multiple ligands are often attached to a single NP resulting in behavior that is complex. Whether or not ligands remain tethered to particle surfaces depends not only on the primary chemical, electrical, photo, and magnetic interactions between each component, but also on concomitant secondary reactions and by-products. Ligands can be selected on the basis of optimizing the intended functioning for the NP. For instance, ferro-magnetic NPs often have surface ligands that promote uptake by cells via a surface receptor. Such ligands include insulin, transferrin, lactoferrin, folic acid, and pullulan [15]. 

Noble metal NPs commonly are equipped with surface ligands composed of thiols, amines, carboxylic acids, phosphines or disulfides [43] because these ligands can alter and optimize NP functioning. The organosulfur compounds of thiols and disulfides spontaneously form strong bonds with the metals and can therefore be used to control the NP size based on ligand concentration. Hydrophobic metal NPs can be made hydrophilic by attaching long chain ammonium ions. Amine ligands increase stability and dispersion when attached to metal NPs. Both amines and long chain ammonium ions reduce the toxicity of NPs. In contrast to amine ligands, which improve dispersability of the NPs in tissues, some ligands are designed so as to have enhanced bonding between different NP components which lengthens the time the NP remains an intact entity within the targeted tissues. These NPs have different surface moieties, which provide multifunctional adsorption and therefore enhance the NP stability during physiochemical interactions. Multifunctional adsorption is created through interactions of negatively charged carboxylate groups with the metal NP and chemical functional groups via deprotonation of the carboxylic acid ligand. In contrast, metal-phosphine ligand bonds are weak and unstable but are used to introduce an NP into the body that will swap a weakly bonded functional group for another moiety available *in situ* that forms a compound through stronger bonding. For example, in a study that compared the effects of differently charge functional ligands on the same Au NP determined that negatively (2‒) charged and the positively (3+) charged ligands induced behavioral abnormalities and reduced survivorship of zebrafish embryos, while the identical Au NP designed with a neutrally charged ligand had no effect [44]. Thus, metal NP properties can be modulated to obtain desired properties through attachment of different surface ligands, but those desired properties may also produce accompanying detrimental effects on exposed cells and tissues. 

Metal oxide NPs offer optical and magnetic capabilities which are often utilized for biomedical applications. Carboxylic acids, phosphonates or silanes are often used to modify the surface of metal oxide NPs. Phosphonate-metal oxide bonds are stable in water, suggesting that NPs with these constituents may have longer half-lives in organisms than more weakly bonded nanomaterials. Silanes are the most popular ligand for metal-oxide NPs because they can support numerous functional groups. Alkoxy- and chlorosilanes are so reactive that they do not require catalysts or water to interact with hydroxyl groups of the metal oxide surface. However, metal oxide NPs modified with these ligands can produce alcohol or hydrochloric acid which can alter or degrade the NP, or harm host tissues [43]. The biotoxic impacts of ligands are not well understood as a factor separate from the surface effects *in toto* in most toxicological studies. However, since ligands are integral to functioning and targeting of NPs in biomedical applications, their effects need to be explored more fully. 

Proteins are also engineered onto the surface of NPs used in medicine. Biologically relevant proteins are anchored to NP surfaces directly or via ligands to improve the performance of an enzyme. For instance, the curved surface of the NP increased stability of lipases derived from *Candida rugosa* and *Pseudomonas cepacia,* at low pH over a wide range of temperatures, compared to non-nanosized structures tested [45]. Enhanced stability and enzymatic activity have also been reported for proteins attached to NPs of gold, silica, and other nanomaterials. These observations could provide the basis for nano-based technological advances for the pharmaceutical, and medical fields [46].

#### Repression of Pro-inflammatory Responses

There are several biological effects induced by NPs that merit further investigation. Although exposures to some NPs may promote oxidative stress and pro-inflammatory responses, other NPs can suppress the normal inflammatory reaction to LPS, an observation that is rarely the focus of discourse. In cells exposed to LPS, platinum NPs can prevent the phosphorylation of ERK1/2, Akt, and IkB-a, and thereby impede the transcription of NFκB [47]. Gold NPs similarly inhibit NFκB and also IFN-β/STAT1 signaling pathways [48]. In murine macrophage-like cells, amorphous silica and superparamagnetic iron oxide (SPIO) NPs interfered with macrophage transition from a pro-inflammatory to a non-inflammatory activation state [49]. The properties of NPs that mediate these responses remain unknown.

## 3. Modeling of NP Interactions with Molecules

### 3.1. Redox

Efforts to characterize the relationships between NP properties and toxicity are underway. These efforts utilize quantitative structural activity relationship (QSAR) modeling. The goal of QSAR modeling is to develop predictive paradigms based on incorporation of chemistry and biological toxicological data. Experimental concepts that link key NP measurements and characterizations to their fate, distribution and effects on tissue are used to construct suites of NPs and test their effects on cells in an iterative process. Eventually, experimental properties (or descriptors) and models are tested on larger, and more diverse groups of NPs [50,51,52]. Thus far, the major modeled mechanism identified by which metal or metal oxide NPs alter host biomolecules molecules is based on redox reactions, described below [4,14,50,53,54,55]. Properties of the liquid environment also affect the cytotoxic activities of NPs in complex systems, but are not addressed by the computational approaches described below because there is a lack of published experimental data for many nanomaterials. For instance, bulk chemical composition, pH, conductivity, viscosity, refractive index, density, and ionic strength exert effects. Additional biological molecules in the dispersant solution contribute to the complexity of testing conditions that need to be taken into account.

#### 3.1.1. Redox—NPs Reduce or Oxidize Host Targets

In addition to the models of colloid behavior (DLVO and XDLVO theory) discussed above, another model called density functional theory (DFT) describes the ability of metals and metal oxides to interact with potential energy available in the chemical bonds of biomolecules. DFT is a quantum mechanical model commonly used in computational physics and chemistry to compute and map the electronic density of a system. With DFT, any chemical system can be described by the electronic chemical potential (the probability of electrons to escape from equilibrium) and absolute chemical hardness (the reluctance to transfer charge, represented by the band gap in molecular orbital energies). Recent research has proposed that semiconductive metal and metal-oxide NPs can act as catalysts of redox reactions, or as electron donors or acceptors [4,53,54,55,56]. When energy levels of the valence (Ev) and conduction (Ec) bands of metals or metal-oxides are similar to energy levels of the Ev and Ec bands in biological molecules, electron transfer is possible. The top of the valence band is the highest level that has completely filled electron positions, whereas the bottom of the conduction band is the first level that has unoccupied electron positions. The calculated Ev and Ec band energy levels are adjusted for pH effected isoelectric points of zeta-potential measurements of the solution [50]. If the energy level of the aqueous redox pair is closer to the Ec of the metal oxide, but slightly higher, then the electron will be donated from the biological substrate (which will be oxidized) to fill the hole in the unoccupied spot of the oxide’s conduction band (which will be reduced). If the aqueous redox potential is closer to the oxide’s valence band, the biological substance will accept the electron and be reduced. The biological potential energy or redox potential range is −4.12 to −4.84 eV [50,56]. NPs with the potential for electron transfer with biological substrates are shown in Table 1. 

#### 3.1.2. Photo-Excitation—A Redox Mechanism Involving UV-Activated NPs

Photo-excitation (UV adsorption) can provide energy for an electron to jump from the valence to the conduction band which will reduce dioxygen to reactive superoxide ions, which in turn can readily reduce hydrogen sources in biological molecules [50,54]. Certain oxides, such as titanium dioxide, are susceptible to UV activation, so this mechanism is relevant for workers exposed to aerosols of titanium.

**Table 1:** Metal oxide nanomaterials with a high or low potential for electron transfer with biological substrates, based on the estimated energy levels of their conduction bands and valence bands [53,56].

Metal oxide potential for electron exchange with biological substrates ^a^						
High likelihood ^b^		Low likelihood ^c^				
Ag_2_O	Mn_2_O_3_	Al_2_O_3_	Eu_2_O_3_	La_2_O_3_	NiO	Ti_2_O
CdO	MnO_2_	As_2_O_5_	Fe_3_O_4_	Li_2_O	PbO	Ti_2_O_3_
Co_3_O_4_	MoO_2_	BaO	Ga_2_O_3_	Lu_2_O_3_	Rb_2_O	V_2_O_3_
CoO	Ni_2_O_3_	BeO	Gd_2_O_3_	MgO	Sb_2_O_3_	V_2_O_5_
Cr_2_O_3_	PbO_2_	CaO	GeO	MnO	Sb_2_O_5_	VO
CrO_2_	Ta_2_O_5_	CeO_2_	GeO_2_	MoO_3_	Sc_2_O_3_	WO_3_
Cu_2_O	Ti_2_O_3_	Ce_2_O_3_	HfO_2_	Na_2_O	SiO_2_	Y_2_O_3_
FeO	TiO_2_	CrO_3_	HgO	NbO	SrO	Yb_2_O_3_
Mn_2_O	WO_2_	Cs_2_O	Ho_2_O_3_	NbO_2_	Tb_2_O_3_	ZnO
		Dy_2_O_3_	K_2_O	Nd_2_O_3_	TiO	ZrO_2_
		Er_2_O_3_				

Notes: ^a^ Discrepant results are omitted from Table 1; [53] concluded that CuO, Fe_2_O_3_, In_2_O_3,_and SnO_2_ could participate with redox reactions, while [56] disagreed. ^b^ High likelihood of electron transfer between the Ec and Ev bands of the metal-oxide and biomolecules. ^c^ Low likelihood of electron transfer between the Ec and Ev bands of the metal-oxide and biomolecules.

#### 3.1.3. Fenton’s Reaction—A Redox Mechanism that Produces Oxide Radicals via Iron and Copper

Fenton’s reaction involves iron, copper and other transition metals in combination with hydrogen peroxide to generate oxide radicals in a two-step reaction. Many biological processes generate hydrogen peroxide which can oxidize ferrous iron (II) in NPs of magnetite (Fe_3_O_4_) to ferric iron (III) in maghemite (Fe_2_O_3_) (described in Equations (1) and (2)). The redox potential for the dissolution of H_2_O_2_ is estimated at 5 eV, close to the valence band value for magnetite, 5.7 eV, supporting electron transfer between the two [50]:

Fe^2+^ + H_2_O_2_ +H^+^→ Fe^3+^ +OH^•^ +H_2_O (1)

Fe^3+^ + H_2_O_2_ → Fe^2+^ +HOO^•^ +H^+^ (2)

The products in Equation (1) can also react with hydrogen peroxide (Equation (2)) creating a net gain of two oxygen radicals (a hydroxyl radical in reaction (1) and a superoxide radical in reaction (2). These highly reactive radicals can oxidize most organic and inorganic compounds. A similar reaction is generated from other iron minerals as well; Fe^0^ can be oxidized to magnetite and lepidicrocite [4]. Despite the potential drawbacks associated with NPs composed of metals susceptible to Fenton’s reaction, they are commonly used because of their magnetic properties, essential for medical diagnostic imaging. 

### 3.2. Modeling NP Interactions with Molecules-Chemical Instability

Another mechanistic model incorporates thermodynamic and kinetic theories to estimate the chemical stability and structure of NPs. Seventeen metal oxide NPs ranging in diameter from 15–90 nm were tested with this model to predict the lethal effects they produced. NP toxicity was defined by the equivalent molar concentration of NPs that produced a 50% reduction (EC_50_) in *E. coli* viability [57]. Instability of the metal oxide was quantified as the enthalpy of formation of a gaseous cation (∆H_ME+_) that had the same oxidation state as the NP solid. The relationship between cytotoxicity and NP instability (∆H_ME+_) was defined as:

log(1/EC_50_)=2.59 – 0.50*∆H_ME+_ (2)

Enthalpy is defined in thermodynamics as internal energy and pressure multiplied by the volume of the system. Internal energy is comprised of the random motion of molecules in the material in the system or in the chemical makeup of the material. Energy is conserved, but can be transformed into other forms of energy (internal, kinetic or potential), and move from a system to its surroundings. The enthalpy of formation of a gaseous cation from a metal oxide solid combines two mechanisms that contribute to metal oxide cytotoxicity: (a) lattice energy, which addresses the dissolution of a cation from the solid state omitting redox reactions, and (b) electronic properties of the valence and conduction bands which contributes to its redox capabilities [57]. This model is based on density functional theory (DFT), similar to the first mechanism proposed by Burello and Worth [50] but requires only a single fitting parameter (enthalpy of formation of a gaseous cation) to estimate toxicity. 

Statistical analysis between the observed linearly related *in vitro* log 1/EC_50_ toxicity values and the nano-QSAR model’s predictions from the validation and training sets produced a squared regression coefficient of R^2^ = 0.85; a cross-validated regression coefficient Q^2^_CV_ = 0.77; and an external validated regression coefficient Q^2^_ext_ = 0.83. These correlations can be considered moderate to good for predictive reliability under the limited model. The negative values for lattice energy of the solid, and the positive values for the enthalpy of formation of a gaseous cation (∆H_ME+_) both increase with increasing cation charge reflecting the higher energy costs required to detach more electrons. A decrease in metal oxide toxicity correlated with an increase in number of cation charges. Another investigation that linked the toxic effects of seven metal oxides on *E. coli* corroborated this phenomenon [58]. Interestingly, in both studies, NP particle size differences did not exert any discernible effect on response. 

### 3.3. Modeling NP Interactions with Molecules—Adsorption Indices

The interaction of amino acid residues of proteins and NP chemical functional groups in a physiological milieu has been characterized by combining contributory adsorption indices for several parameters, and is called the biological surface adsorption index (BSAI) [42]. These five parameters include the molecular force of lone pair electrons, dipolar and polarizability potential, solute acid/base measure, London dispersion forces, and zeta-potential. Many of these intermolecular forces are relevant to DLVO and XDLVO theory, but measured at a nano-scale. This method involves measuring the competitive adsorption of probes of small molecules located on the NP surface which approximates how amino acid protein residues of the corona react with the NP. Investigating the molecular interactions of biomolecules with the NP surface could be used to model corona formation and activity. In addition, this model can help evaluate biodistribution characteristics for cellular uptake of drugs and chemicals, including kinetic and adsorption rates and electrostatic interactions. Although extrapolations of small case studies to more generalized applications may be complex, the advantages of this mechanistic model for measuring bio-nano effects is that it can be suitable for quantifying any type of nanomaterial in a variety of matrices [50].

### 3.4. Modeling NP Interactions with Molecules—Dissolution

Several studies have reported that moderate to highly soluble metals and metal oxides can dissolve with or without oxidation and reduction in a mechanism that is not yet understood. Ions that are taken up by cells and sequestered in macrophage lysosomal compartments dissolve rapidly due to the low lysosomal pH, and can destabilize the lysosome membranes. The accumulation of Zn^2+^ ions in lysosome and caveolae are associated with oxidative pro-inflammatory responses, organelle clumping, intracellular Ca^2+^ and cytochrome c release, mitochondrial permeability transition pore opening (likely related to Ca release) with subsequent apoptosis and necrosis [34,56,59]. Oxides implicated in this type of chemical breakdown include CuO, ZnO, Mn_3_O_4_ and Co_3_O_4_ and possibly SiO_2_ [4,34,56,59]. Factors that increase the rate of dissolution and toxicity include elevated metal solubility, low pH, the presence of amino acids and peptides, surface/volume ratios, surface curvature, and surface roughness [4]. Lead sulfide NPs similarly exhibit decomposition and precipitation upon oxygen exposure [60]. Two lead sulfide NPs of comparable size and core composition were functionalized with different surface ligands, sodium 3-mercaptopropane sulfonate (MT) or sodium 2,3-dimercaptopropane sulfonate (DT). The PbS-MT NPs induced 100% mortality in zebrafish embryos, whereas the PbS-DT NPs resulted in less than 5% mortality at the same concentration due to faster disintegration of the MT surface ligand with greater leaching of the decomposing PbS toxic core.

## 4. Modeling the Biological Effects of NPs

Attempts are being made to systematically model the biological effects of NPs and correlate these effects with NP structural features. In some studies, NP construction is linked to specific biological properties, such as preferential uptake by specific tissues. For example, a NP library of common magnetofluorescent cores conjugated to 146 different types of small molecules was synthesized and approximately 60 copies of each small molecule were attached to a 38 nm diameter NP. [61]. Surface groups were selected for their hydrophilicity and their tendency not to bind to proteins. The surface groups consisted of amines, alcohols, carboxylic acids, sulfhydryls and anhydrides. Overall, the NPs had high magnetic relaxivity, which promotes biocompatibility. The library was tested by high throughput screening for relative uptake in five different cultured cell lines in order to identify a NP that targets pancreatic ductal adenocarcinoma cells. The investigators identified fourteen fabricated NPs that were taken up by the adenocarcinoma cells at higher rates than other NPs. Two of these fourteen NPs were also taken up poorly by control cells (macrophages and endothelial cells). These two NPs had the same magnetofluorescent core material, but had distinct surface moieties (isatoic anhydride and 5-chloro-isatoic anhydride) l [61]. In mice, both NPs accumulated in pancreatic tumors. Accordingly, higher uptake in pancreatic cells was observed, as compared to the low uptake in liver, lung, muscle and other organ tissues. Although this approach proved successful, and each designed NP exhibited a unique biological response, the specific structural properties of these NPs that contributed to their differential cell uptakes remain unknown. 

Another study also examined the effect of slight differences in NP design on cellular response [62]. Shaw and colleagues varied NP concentration in addition to core material, and surface moieties. The effects of these variables on ATP content, reduction equivalents, mitochondrial membrane potential, and apoptosis were quantified in high throughput assays. Physiological responses were averaged across four cell types (endothelial, smooth muscle, monocytes and hepatocytes). The resulting combination of four assays, in four cell types, measured at four doses produced a 64-biological vector. Correlations between NP features and biological response were obtained using different algorithms including classification and regression trees, k-nearest-neighbors and weighted voting. The biomarker with the strongest correlation between NP features and activity was apoptosis. The averaged physiological responses incurred by each designed NP was distinctive, even when two constructed NPs differed only by a single diastereomeric modification in a similar peptide. Two NPs with the same Fe_3_O_4_ cores and polyvinyl alcohol (PVA) coatings, but slightly different surface modifications (L-arg_8_-COOH * vs.* L-arg_7_-COOH) produced singular biological responses. A pair of NPs that differed in their core compositions of Fe_3_O_4_ and Fe_2_O_3_ but had similar PVA coatings and protamine and rhodamine surface moieties could also be distinguished by the cellular responses they stimulated [62]. In summary, both studies [61,62] illustrated a trend reported by numerous studies: the biological responses induced by NPs are determined by contributions of the core composition as well as the surface modifications.

The effect of different NPs’ core compositions on key cellular signaling pathways has also been explored. In one study with macrophage-like cells, the dosage effects on cellular transcription of seven metal and metal oxide NPs with diameters ranging from 8–20 nm were monitored [52]. The investigators utilized self-organizing maps to group NPs into five clusters based on their effects on reporter gene expression for ten major signaling pathways. A consensus index ranging from 0 to 1 provided a statistical measure of the validity of clustering for each identified group. Two clusters, one consisting primarily of platinum (Pt) or zinc oxide (ZnO) induced a cell cycle regulator, (E2F), early (3 and 6 h) and a DNA damage reporter, p53, late (12 h). ZnO and Pt NPs also reduced expression of the PKC/Ca^2+^ pathway possibly due to the initiation of apoptosis, which is also triggered by p53. ZnO NPs also decreased signaling pathways associated with inflammation (NFκB, MAPK/JNK, and MAPK/ERK) at 3–6 h post-exposure. A third NP cluster increased expression of reporters of inflammation and cell cycle (E2F). This NP cluster consisted of moderate to high dosages (≥25 μg/mL) of NPs of SiO_2_, Al_2_O_3_, Fe_3_O_4_ and moderate to low dosages (<12.5 μg/mL) of NPs of Au, Pt, and ZnO. The promotion of inflammation induced by ROS generation by this NP cluster is consistent with previous observations. These observations indicate that NP core composition affects cellular physiology. 

One research group has produced a comprehensive computational QSAR modeling effort by combining causative mechanistic descriptors unique for each NP structure with singular cellular responses induced by each NP design [51,63]. Four parameters (descriptors) were sufficient to characterize all NPs tested; the QSAR model incorporates mechanisms that address potential energy (R1 and R2 magnetic relaxivities) and two empirical factors that are important regulators of NP structure and its induced effects, size (diameter) and zeta-potential. Relaxivity is a term derived from nuclear magnetic resonance, and has only been recently used to analyze NPs. Relaxivity is defined as the time required for nuclei to return to their equilibrium state and orientation after a magnetic force field, previously applied, has been switched off. R1 and R2 are the times required to re-establish equilibrium in the directions parallel to (longitudinal) and perpendicular (transverse), respectively, to the axis of magnetization and unique for each substance [64].

The proposed QSAR model is singular because it links a predictive mechanistic model to observed biological activity reported by other research groups. Although two previous studies [61,62] detailed how unique biological responses can be coincident with singular constructions of NPs, they did not provide a causal physico-electric chemical model of NP structure that would provoke these responses. Fourches *et al.* used the data from both these studies [61,62] to test their newly developed parameters, algorithms and methods of their model and to compare the results. 

Fourches *et al.* discovered that their four-parameter model could adequately differentiate between each of the 51 unique NP constructions composed of different cores and surface groups examined by Shaw *et al.* However, the model of Fourches *et al.* could not predict a distinct toxicity signature for each NP unless all the 64 biological data responses for each NP were averaged into a single response. Fourches *et al.* also discovered that inclusion of an additional NP descriptor (surface lipophilicity) better captured the variability in NP biological responses [61]. The lipophilicity of the surface modifiers correlated with NP induced endocytosis and toxicity for the set of 109 constructed NPs that had similar cores with different surface small molecule moieties [61]. Although some criticism of the studies by Fourches *et al.* debates whether magnetic relaxivity is a correlative, rather than a causative mechanism of NP behavior [65], overall Fourches *et al.* correctly predicted the biological outcomes for classes of NPs examined by Shaw *et al.* and Weisslander *et al.* 73% of the time [51]. 

Relaxivity was also used in a recent study of multifunctional therapeutic magnetite NPs constructed with hydrophobic and hydrophilic cores. Structural dissimilarities were reflected in differences in their longitudinal and transverse relaxivities. Although the transverse relaxivities of the two types of cores correlated with size and compositional differences, NPs with hydrophilic cores measured more than twice that of the hydrophobic core NPs. One possible explanation for the elevated relaxivities was that the diffusion of water into the hydrophilic cores produced high magnetic field gradients [66]. 

## 5. The Future of Modeling

While modeling NP induced biological effects has progressed in the last ten years, new methodologies that can predict NP properties are being discovered and need to be incorporated into QSAR and QSAAR modeling efforts. Such models will be used in the regulation of industry to insure public health and safety and promote safer designs of nanomaterials. Thus, it is essential that the predictive models encompass the full spectrum of NP behavior. The structures of many NPs remain unknown, and there is no universal codification of structural, chemical, and electrical properties of nanomaterials. Models therefore have generally relied upon descriptors (mathematically represented parameters) rather than empirical features and include metal ionization, zeta potential, relaxivity, heat of formation, orbital energies and redox potentials. It has been noted that certain physicochemical differences exert influence on these descriptors (*i.e*., size, lipophilicity, surface area, and surface modifications). Future structure-activity models will need to quantify how these factors interrelate to develop a more comprehensive, robust causative model that can be readily tested on diverse nanomaterials. There are some methods available, such as quantum chemistry, molecular dynamics, and spectral analyses that may provide insights into NP mechanisms and properties [65], but an entirely novel analytical framework needs to be developed for nanomaterials.

Chemical structural activities and their toxicological effects have been characterized and understood over many years, but cannot be incorporated into models of NP behavior. Chemicals have been well tested for biological genetic mutation, toxicological, reproductive, developmental, and carcinogenic effects using assays designed for exploring the full spectrum of behavior related to pre-identified relevant concentration ranges. The problem with our understanding of NPs is that neither their structures, properties, nor effective concentrations are known. However, we can still learn from examining the successes and shortcomings of chemical QSAR modeling, and apply them to NP QSAR modeling efforts, and reviewing the limitations of acellular, cellular and whole organism testing of chemicals may provide insight into designing improved biological testing for nanomaterials. 

The E.P.A.’s Toxcast program uses high throughput screening (HS) and high-content screening (HCS) methods combined with 467 *in vitro* assays to predict chemical toxicity. The Toxcast Phase II program (http://www.epa.gov/ncct/toxcast/) also includes NPs, but results have not been published as yet. Toxcast chemical assay results are analyzed with computational algorithms to predict a chemical’s potential for inducing toxicity pathways via specific modes of action, such as disruption of microtubule assembly, oxidative phosphorylation, and platelet aggregation. A performance review of Toxcast’s Phase 1 analysis of 309 chemicals, primarily commercial pesticide products, included evaluating the predictive capabilities of 292 acellular bioassays that included enzymatic and binding assays for G-protein coupled receptors, cytochrome P450 mono-oxygenase enzymes, kinases, phosphatases, proteases, ion channels, nuclear receptors, transporters [67]. Shortcomings of Toxcast’s toxicity forecasting stemmed from discrepancies between acellular and whole organism results. For instance, multiple cases of interactions between chemicals and nuclear receptors that occur in whole organisms were not detected *in vitro*. A chemical sample group of direct-acting cholinesterase inhibiters, for example, frequently tested negative (38%) in cell culture, and positive (94%) in whole animal tests. Furthermore, for indirect-acting anticholinesterase inhibitors, over half of the acellular assays tested negative, in contrast to the overwhelming positive whole animal responses. These divergences may be explained by the complexity of nuclear receptor activation which may involve partial or indirect agents, or stabilized by extrinsic or intrinsic factors that may not be recapitulated in cultured cells. Other chemical compounds, such as fungicides, have multiple cellular targets, each of which may or may not be present in a given cell line, resulting in under- representative responses observed in the suite of assays. Finally, many pesticides and fungicides (*i.e*., chlorpyrifos-ethyl/chlorpyrifos-oxon, malathion/malaoxon) are modified within cells or tissues, processes that may not occur within cell lines, so the inactive parent compound tests negative in acellular systems, whereas the bioactive metabolite exerts its effects and is detectable only in whole organisms [67]. Thus, there are many complex reasons why acellular, and cell culture results may not reflect animal data. Although many researchers and regulatory agencies are promoting the use of these tests with QSAR modeling to forecast NP toxicity and regulate accordingly [65], it seems that a reliance on their accuracy could be foolhardy.

## 6. Certain Cellular and Whole Animal Testing May Not Accurately Represent the Disease Process

Toxicity and carcinogenicity analyses of larger-sized chemicals have revealed the limitations of cell culture and animal models for representing disease processes, limitations that may well apply to NPs. Two papers did critical analyses of various genetic toxicity (genotox), and reproductive and developmental toxicity tests (reprotox) as indicators for cancer development reported by rodent *in vivo* bioassay results (rcbioassays) and establishing common *in silico* surrogate physiological endpoints. With statistical and cross validation modeling, each of the genotox and reprotox tests were ranked by the correlation indicator (CI) which averaged the specificity and sensitivity of the test and its predictability for identifying positive inducers of cancer [68,69]. The researchers performed QSAR analysis of 63 different genetox and reprotox tests with various toxicological endpoints addressing gene mutation, clastogenicity, DNA damage, cell alteration, and toxic effects in reproduction, development, and fetal growth/behavior. Analysis demonstrated that a consensus existed between the genetox and reprotox tests that could differentiate between two types of carcinogens. Type I carcinogens had 2 or more positive genotoxic or reprotox tests and usually could be identified through *Salmonella* mutation assays. Type II carcinogens tested as inactive or positive in only one of the genetox/reprotox battery of tests, and usually were not detected by the *Salmonella* gene mutation test. The researchers also noted that the genetox array of tests seems to sense cancer mechanisms and pathways separate from those of the reprotox suite of tests, and both are required to detect the full range of inducers for rodent carcinogenesis. The *Salmonella* mutation assay has been determined by the National Toxicology Program to have good specificity, but low sensitivity, and can only detect less than half of carcinogenic chemicals, because it is reactive to only a subset of all the multiple mechanisms and signaling pathways that instigate tumorogenesis in rodents. However, it was notable that *in vivo* tests comprised 88% of the individual tests that were good indicators of carcinogenesis, and 42% of the individual tests that were poor indicators, so it is clear that although whole animal tests overall may produce more reliable results, some *in vivo* tests are better predictors than others [68]. In conclusion, the researchers surmised that the addition of eight specific genetox and reprotox assays to the *Salmonella* mutation assay could adequately project rodent carcinogenesis as indicated by the rcbioassays. Conducting these types of QSAR modeling and evaluative studies should be done for NPs to assess which assays would provide the best information of discerning the signaling pathways and mechanisms by which toxicity progresses in exposed organisms. Retrospective analyses of chemical toxicity studies suggests that selecting a suite of whole animal tests for researching NP-induced toxicity would provide the most comprehensive and reliable forecasting.

### Important Aspects of Whole Animal Testing

Progressive techniques in automated processing has escalated the use of small invertebrate animals for testing disease processes in recent years These assays offer advantages of having well-characterized genetic and developmental biologic systems that can be tested with lower costs and in less time than mammalian studies due to their adaptation to high-throughput processing [70]. Exposure of fruit flies (*Drosophila melanogaster*) to silver NPs induces genetic evidence of oxidative stress, membrane damage, DNA damage, mitochondrial damage and subsequent apoptosis, based on biomarker expressions similar to those used in vertebrate studies [71]. Inherited mutations were also observed in *Drosophila* exposed to gold NPs [72]. Roundworms (*Caenorhabditis elegans*) have exhibited developmental defects upon ingestion of silica NP including episodes of failed egg-laying usually noted in senescent worms with reproductive organ degeneration [73]. Embryonic zebrafish have been used to test 1060 compounds of phase 1 and 2 U.S. EPA Toxcast list in a high-throughput screening method to evaluate multi-dimensional *in vivo* effects [74]. An example of some of the results demonstrated that the set of selected developmental endpoints in embryonic zebrafish could accurately predict 78% of the neurotoxicants tested, including malathion, but not chlorpyrifos. Furthermore, embryonic zebrafish are transparent in the first 48–60 h of development which allows tracking of any chemically induced abnormalities of organ development easy to visualize, an advantage over whole animal rodent assays [22]. There are also many mutant and transgenic lines available [22]. The option of expanding the use of these types of whole animals for testing how NP exposures affect developmental stages of cell differentiation, immunological, neural and reproductive function could be investigated and may be a worthwhile investment, and an alternative to slower rodent/ mammalian whole animal studies. 

The proposal that the activities of NPs in organisms can be predicted by combining structure parameter-activity relationships with *in vitro* (cellular and acellular) screening [65] needs to be reexamined. The advantages, as compared to reliance on whole organism testing, include reduced cost, effort and time expenditure. However, there is a real need to put the cellular/acellular/whole animal test results into a more realistic context. There are problems with: (1) applying biochemical modeling to cell culture or whole animal results, (2) cell culture not being predictive of whole animal results, and (3) interpolating whole animal results across species to humans. Whole organisms are much more complicated than cultured cells, clearly illustrated by the difficulty of extrapolating physiologically relevant dosing levels from cell culture to animals. Responses can differ significantly; for example, in a recent study [75] the toxic effects of five NPs in rats upon instillation did not correlate with reactions observed in cultured rat L2 lung epithelial cells, macrophage cells, nor co-cultures of the two. Expression in lung cell culture of pro-inflammatory cytokines TNF-α and IL-6 did not correspond either to each other, or to the whole animal reaction. Recruitment of inflammatory cells into the lung is an important pathogenic process that cannot be mimicked within cell culture and may have contributed to the observed discrepancies. In addition, NP cytotoxicity in the different cell cultures and in the rats did not correlate well either. There is a real concern that other published cell culture studies will not translate to NP behavior in whole animals. 

## 7. Conclusions

Understanding and modeling the mechanisms in which NPs induce biological activity in exposed hosts has progressed dramatically in the last decade. But more explicit research linking causative mechanisms of energy transfer between biomolecules and NPs is needed. In addition, although most models concentrate on modeling toxicity endpoints, additional non-fatal but damaging cellular and organismal consequences need to be explored. 

In order to understand and regulate potential adverse effects of metal and metal-oxide NPs, significant advances need to be researched in systematic ways. Accurate and uniform testing for the relevant physicochemical properties of NPs in their pristine manufactured condition that determines their behavior needs to be established. In addition, testing for their activity under different environmental conditions and their pharmacokinetic interactions, biotransformations and emissions needs to be instituted. Valid inferences need to be developed between their toxic, genomic and proteomic effects in order to create models that accurately represent realistic outcomes in complex systems. These should be founded on modeling efforts derived from cogent and substantive *in vitro*-*in vivo* extrapolations of biological tests. 
